# Production and characterization of Zn- and Cu-doped Y2O3–Al2O3–SiO2 (YAS) glass microspheres

**DOI:** 10.55730/1300-0144.5888

**Published:** 2024-09-08

**Authors:** Fatma ÜNAL, Batur ERCAN

**Affiliations:** 1 Department of Biomedical Engineering, Faculty of Engineering and Natural Sciences, Samsun University, Samsun, Turkiye; 2 Vocational School of Technical Sciences, Samsun University, Samsun, Turkiye; 3 Department of Metallurgical and Materials Engineering, Faculty of Engineering, Hitit University, Çorum, Turkiye; 4 Department of Metallurgical and Materials Engineering, Faculty of Engineering, Middle East Technical University, Ankara, Turkiye; 5 BIOMATEN, Center of Excellence in Biomaterials and Tissue Engineering, Middle East Technical University, Ankara, Turkiye; 6 Biomedical Engineering Program, Middle East Technical University, Ankara, Turkiye

**Keywords:** Yttrium aluminum silicate, glass microspheres, sol-gel derived method, radioembolization

## Abstract

**Background/aim:**

Y_2_O_3_–Al_2_O_3_–SiO_2_ (YAS) glass microspheres are currently used in radioembolization treatment. However, abscess formation can occur following this treatment. This study aims to endow YAS glass microspheres with antibacterial properties to address the abscesses forming in patients after radioembolization treatment.

**Materials and methods:**

In this study, undoped YAS glass microspheres and those doped with antibacterial agents zinc (Zn) and/or copper (Cu) were successfully fabricated using a sol-gel derived method.

**Results:**

After heat treatment, the microspheres exhibited an amorphous structure. Additionally, the incorporation of Zn and/or Cu dopants did not alter the patterns observed in the X-ray diffraction analysis. Fourier transform infrared spectroscopy analysis detected Si–O–Si, Al–O–Al, and Y–O band vibrations within the structure. The presence of Zn and Cu dopants was confirmed through X-ray photoelectron spectroscopy analysis. Scanning electron microscopy revealed that all samples possessed a regular microsphere morphology, with average particle sizes ranging from 6 to 50 μm. These average particle sizes were further confirmed using a mastersizer.

**Conclusion:**

The antibacterial agent-doped YAS glass microspheres show promise in combating infections that occur following radioembolization treatment.

## Graphical Abstract

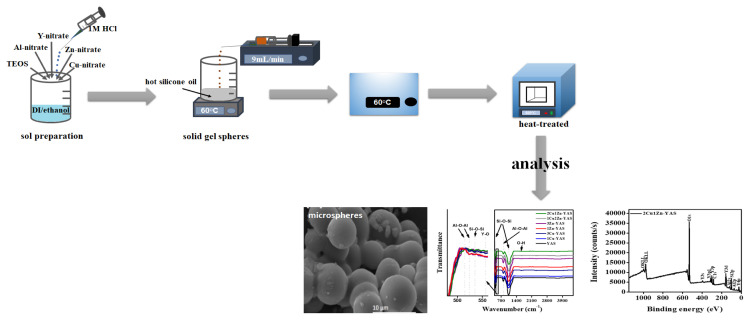

## 1. Introduction

Surgical intervention is a commonly employed approach for treating cancer. However, postsurgical recovery of the dissected organ may not be complete, which poses a significant concern, particularly when the removed tissue is from a vital organ such as the liver. To address this issue, a minimally invasive radioembolization technique designed only to target and destroy cancerous cells was developed in the mid-1980s [1–5]. This method uses radioactive yttrium aluminum silicate (Y_2_O_3_–Al_2_O_3_–SiO_2_; YAS) microspheres to treat malignant tumors located deep in the body. Compared to surgical intervention, this technique offers several advantages: it requires minimal medical equipment, is noninvasive, and results in faster patient recovery. The procedure involves placing thousands of tiny radioactive YAS microspheres into the arteries that supply blood to the tumor. Once implanted, these microspheres become lodged in the capillary bed of the tumors, allowing for localized irradiation of the cancerous tissue [6–11]. This targeted approach confines irradiation primarily to cancerous cells, minimizing damage to surrounding healthy tissue. The potential use of 17Y_2_O_3_–19Al_2_O_3_–64SiO_2_ (mol%) ceramic microspheres for in situ irradiation of cancers via radioembolization was first reported by Hyatt and Day in 1987 [1]. Subsequent research on this topic was continued by Erbe and Day in 1993 [2]. Today, these ceramic microspheres are widely used in clinics worldwide, saving thousands of lives each year.

Although YAS glass microspheres are currently utilized in radiation oncology, abscess formation following cancer treatment remains contentious. This complication can lead to inflammation, bacterial contamination, and infection. Attasi et al. reported that abscess formation occurred in 98 of 327 patients with hepatocellular carcinoma (190) or liver metastases (137) after undergoing radioembolization therapy [12]. Mascarenhas et al. treated a 58-year-old woman with a gastrin-secreting cell tumor using yttrium-90 radioembolization. Following the treatment, *Escherichia coli* (*E. coli*) bacteria were detected in the patient, prompting the administration of antibiotic therapy [13]. Korkmaz et al. administered radioembolization treatment to a 53-year-old female patient with pancreatic cancer. Unfortunately, the *E. coli* infection that occurred posttreatment led to abscess formation and resulted in the patient’s death [14]. Similarly, Kurilova et al. treated 10 patients with ^90^Y glass microspheres and observed liver abscesses following the treatment [15]. Wong et al. also reported abscess formation after treating liver metastases [16].

In the event of infection, the primary treatment method has traditionally been using antibiotics. However, antibiotic treatment for bacterial biofilms has proven less effective, and bacteria have progressively developed resistance to commonly used antibiotics, such as Methicillin-resistant *Staphylococcus aureus*. This escalating resistance further complicates the fight against infections. Infection remains a significant clinical challenge, prompting extensive research to mitigate or eliminate infection-related risks. It is well-established that employing materials with unique antibacterial properties can reduce infection risk. Additionally, methods such as ion release have been shown to inhibit bacterial resistance and contribute to infection control [17].

Antimicrobial agent-doped materials, including those containing copper (Cu), zinc (Zn), Mg, Ag, Se, and Sr, have been employed in combating bacterial infections [18–25]. Zn and Cu, which are essential trace elements for humans [26–28], are currently utilized in clinical biomedical applications [29–31] due to their antibacterial properties and biocompatibility [18,26,28,32,33]. Unal et al. reported that Zn-doped 58S bioglass effectively inhibited the growth of *S. aureus* and *E. coli* bacteria [19]. Kumar et al. documented the antibacterial activity of Zn-doped S53P4 glass against *E. coli* and *S. aureus* [34]. Additionally, Sánchez-Salcedo et al. observed that Zn-doped bioglass demonstrated antibacterial behavior, specifically against *S. aureus* [35]. Hosseini et al. reported that Cu-doped mesoporous bioactive glasses exhibited antibacterial properties against Methicillin-resistant *S. aureus* bacteria [36]. Bari et al. found that Cu-doped mesoporous bioactive glasses showed antibacterial effects against *E. coli*, *S. aureus* and *Staphylococcus epidermidis* (*S. epidermidis*) [21]. Alasvand et al. documented that Cu-doped glass effectively inhibited the survival of *S. aureus* and *Pseudomonas aeruginosa* (*P. aeruginosa*) bacteria [37]. While numerous studies have explored the antibacterial properties of materials doped with Zn and Cu, research specifically addressing Cu and Zn doping of YAS microspheres is lacking. Therefore, this study aims to synthesize, for the first time, Zn- and Cu-doped YAS microspheres using a sol-gel-derived method. By capitalizing on the antibacterial properties of YAS, the study aims to prevent infection formation in patients, thereby potentially reducing or eliminating the need for antibiotic treatment.

## 2. Materials and methods

### 2.1. Production of YAS glass microspheres

Yttrium (III) nitrate hexahydrate (Y(NO_3_)_3·_6H_2_O), tetraethyl orthosilicate (C_8_H_20_O_4_Si), and copper (II) nitrate trihydrate (Cu(NO_3_)_2_·3H_2_O) were obtained from Acros (Acros Organics, Geel, Belgium). Aluminum nitrate nonahydrate (Al(NO_3_)_3_·9H_2_O) and zinc nitrate hexahydrate (Zn(NO_3_)_2_·6H_2_O) were acquired from Sigma-Aldrich (Sigma-Aldrich Corp., St. Louis, MO, USA). Hydrochloric acid (HCl, 37%) was supplied by Merck (Merck, Rahway, NY, USA). The synthesis of the glass microspheres involved two steps: a) sol preparation and b) microsphere formation. The resulting microspheres had the following composition range: 17 mol% Y_2_O_3_, 64 mol% SiO_2_, 16–19 mol% Al_2_O_3_, 1–3 mol% CuO, and 1–3 mol% ZnO. The detailed chemical compositions of the microspheres are presented in Table 1.

#### a) Sol preparation

For sol preparation, tetraethyl orthosilicate (TEOS) was introduced into a distilled water/ethanol solution (volume ratio of 2:1) and mixed at 300 rpm to facilitate the hydrolysis and polycondensation of TEOS. Based on the desired composition of the microspheres, aluminum nitrate (Al-nitrate), yttrium nitrate (Y-nitrate), copper nitrate (Cu-nitrate), and zinc nitrate (Zn-nitrate) salts were sequentially added to the prepared TEOS solution at 20-min intervals, with continued mixing at 300 rpm. Subsequently, a 1M HCl solution was added to induce sol formation, and the mixture was stirred for 1 h following the formation of the sol.

#### b) Formation of the microspheres

The obtained sol was loaded into a 21G needle syringe and injected into hot silicone oil (approximately 60 °C) at a 9-mL/min flow rate using a syringe pump under magnetic stirring. After injection, the solid gel spheres were separated from the hot oil using hot water, followed by rinsing with petroleum ether and distilled water to ensure complete oil removal. The separated microspheres were then dried at 60 °C for 24 h. Finally, the microspheres were heat-treated at 600 °C for 5 h at a heating rate of 3 °C/min

### 2.2. Characterization of the microspheres

Structural analyses of the synthesized microspheres were conducted using X-ray diffraction (XRD) analysis at a scan speed of 1°/min over the 2θ range of 10–90°, employing a Rigaku X-Ray Diffractometer (Rigaku, Tokyo, Japan). To elucidate the molecular bond properties of the microspheres, Fourier transform infrared spectroscopy (FT-IR) measurements were performed in the 4000–400 cm^−1^ wavenumber range using a Hyperion 1000 IR microscope device in ATR mode (Bruker Optik Gmbh, Ettlingen, Germany). The elemental composition of the microspheres was analyzed by X-ray photoelectron spectroscopy (XPS) using a PHI 5000 Versaprobe instrument, with binding energy (BE) calibrated against the C1s reference peak at 284.80 eV. Morphological analysis was carried out through scanning electron microscopy (SEM) using a Nova Nano SEM 430 (FEI Company). For SEM analysis, the microspheres were mounted on an aluminum holder and coated with a gold-palladium alloy using a Quorum SC7640 high-resolution sputter coater (Quorum Technologies, East Sussex, UK). The particle sizes of the microspheres were determined using a Malvern Mastersizer 2000 (Malvern Panalytical, Malvern, UK), and the specific surface area was measured with a Quantachrome Autosorb-6 surface area analyzer (Anton Paar Quantatech, Boyton Beach, FL, USA). The measurements were based on a liquid dispersion with a refractive index of 1.658.

## 3. Results

### 3.1. Structural analysis

**Figure 1:**
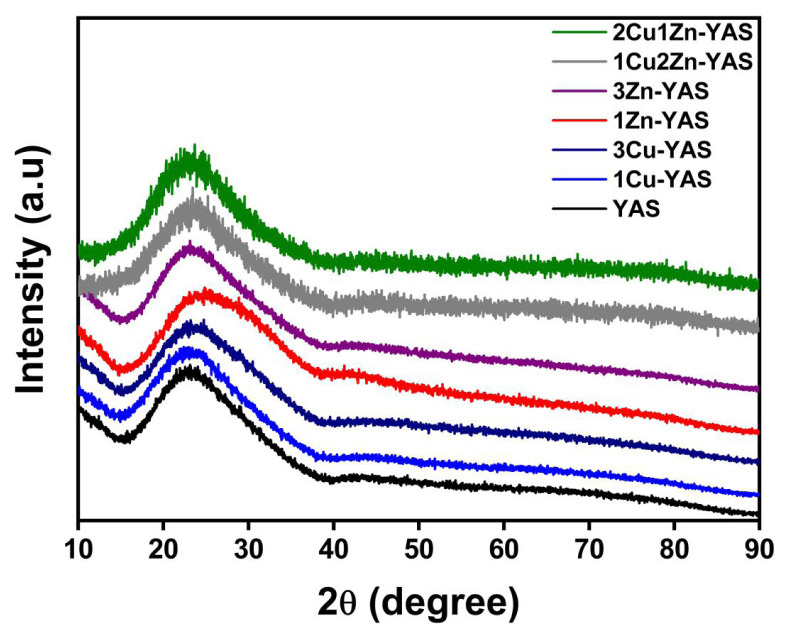
XRD patterns of the YAS, 1Cu-YAS, 3Cu-YAS, 1Zn-YAS, 3Zn-YAS, 1Cu2Zn-YAS, and 2Cu1Zn-YAS microsphere samples.

The XRD patterns of all the microspheres are presented in Figure 1. The microspheres displayed an amorphous structure, characterized by an amorphous hump observed between approximately 2θ = 15° and 40° for all powders. Additionally, incorporating Zn and/or Cu dopant elements did not alter the XRD patterns, indicating that the dopants did not influence the crystallization process.

### 3.2. Chemical analysis

**Figure 2:**
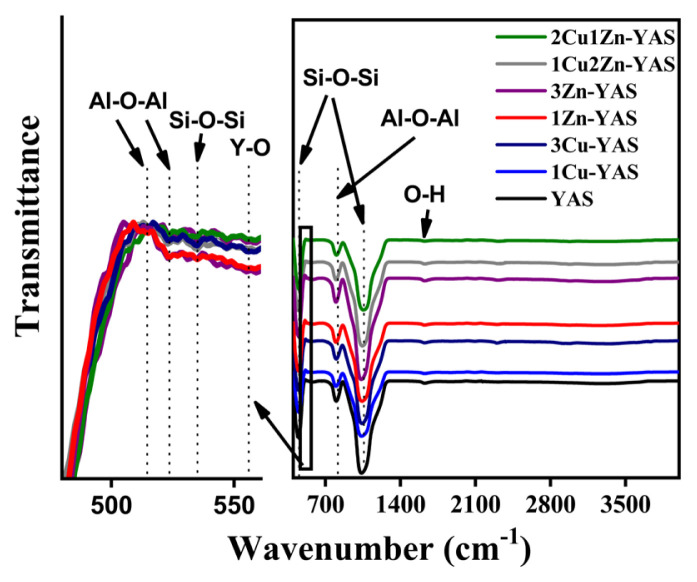
FT-IR spectra of the YAS, 1Cu-YAS, 3Cu-YAS, 1Zn-YAS, 3Zn-YAS, 1Cu2Zn-YAS, and 2Cu1Zn-YAS microsphere samples.

The FT-IR spectra of the YAS, 1Cu-YAS, 1Zn-YAS, 3Cu-YAS, 3Zn-YAS, 1Cu2Zn-YAS, and 2Cu1Zn-YAS microsphere samples are presented in Figure 2. The observed peaks confirm the successful synthesis of the YAS chemistry. The peak around 454 cm^−1^ is attributed to the Si-O-Si bending vibration [38], while the peaks around 535 and 1080 cm^−1^ are associated with the Si–O–Si vibration [39,40]. The peak at approximately 515 cm^−1^ corresponds to the aluminum oxide stress mode [41]. The peaks around 524 and 807 cm^−1^ are assigned to Al-O-Al vibrations [42,43], and the peak at approximately 557 cm^−1^ indicates the vibrations in the Y–O bonds [44]. The peak around 1627 cm^−1^ was attributed to the bending mode of adsorbed water molecules [40,45]. The band assignments from the FT-IR data are detailed in Table 2. The bond vibrations of Zn- and Cu-doped elements could not be detected by FT-IR analysis due to their low concentration in the structure. Therefore, XPS analysis was conducted to confirm the presence of Zn and Cu dopant elements in the microspheres.

**Figure 3:**
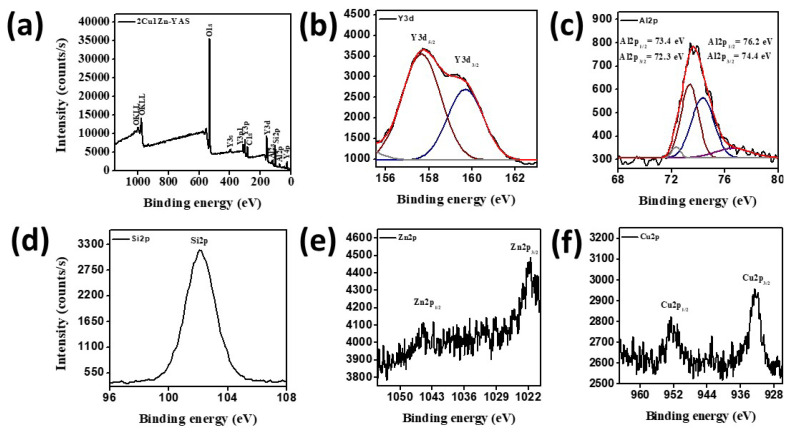
XPS (a) wide spectrum and high resolution (b) Y3d, (c) Al2p, (d) Si2p, (e) Zn2p, and (f) Cu2p spectra of the 2Cu1Zn-YAS microsphere sample.

The elemental composition of the 2Cu1Zn-YAS microspheres was analyzed using XPS. The XPS wide-scan spectrum is shown in Figure 3a and confirms the presence of Si, Al, Y, and O elements. Since the dopant elements were not visible in the general spectrum, high-resolution scans were conducted to verify the presence of these dopants (Figure 3b–f). The high-resolution XPS spectrum for Y3d (Figure 3b) revealed BEs of Y3d_3/2_ at 159.70 eV and Y3d_5/2_ at 157.61 eV [46–48]. The XPS spectrum for Al2p showed BEs of Al2p_1/2_ at 73.4 eV and 76.2 eV and Al2p_3/2_ at 72.3 eV and 74.4 eV (Figure 3c) [49–51]. The XPS spectrum for Si2p exhibited a BE of 102.1 eV, as illustrated in Figure 3d [52]. The high-resolution XPS spectrum for Zn2p (Figure 3e) revealed BEs for Zn2p_3/2_ and Zn2p_1/2_ centered at approximately 1021.9 eV and 1044.9 eV, respectively [53,54]. The high-resolution XPS spectrum of Cu2p (Figure 3f) indicated BEs of 932.2 eV and 952.6 eV for Cu2p_3/2_ and Cu2p_1/2_, respectively [55,56]. Consequently, the XPS analysis confirmed the incorporation of Zn and Cu dopant elements into the structure. The spectral lines and BEs are summarized in Table 3.

### 3.3. Morphological analysis

**Figure 4:**
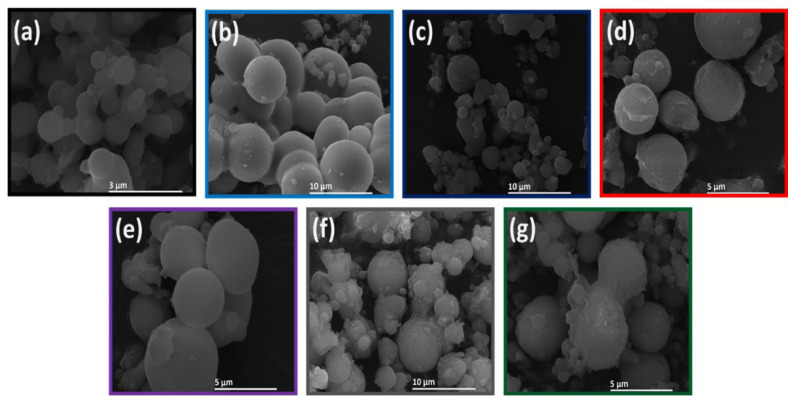
SEM images of the (a) YAS, (b) 1Cu-YAS, (c) 3Cu-YAS, (d) 1Zn-YAS, (e) 3Zn-YAS, (f) 1Cu2Zn-YAS, and (g) 2Cu1Zn-YAS microsphere samples.

SEM images of the YAS, 1Cu-YAS, 3Cu-YAS, 1Zn-YAS, 3Zn-YAS, 1Cu2Zn-YAS, and 2Cu1Zn-YAS samples are shown in Figure 4a–g. The images revealed that all microsphere samples exhibited a regular spherical shape. However, the particles displayed an agglomerated morphology, with particles adhering to one another. The average particle size ranged from 6 to 50 μm, depending on the type and amount of the dopant used. These particle size measurements were also obtained using the mastersizer method and are detailed in Table 4.

### 3.4. Particle size measurement

Table 4 provides the average particle size and specific surface area values for the YAS, 1Cu-YAS, 1Zn-YAS, 3Cu-YAS, 3Zn-YAS, 1Cu2Zn-YAS, and 2Cu1Zn-YAS glass microspheres. It was observed that the average particle size depends on the type and amount of dopant used. The variation in particle size can be attributed to the sol stage, where the silica network (-Si–O–Si-) is formed through the polycondensation of Si–OH groups. This network is subsequently modified by adding Y-nitrate, Al-nitrate, Cu-nitrate, and Zn-nitrate salts, influencing the final particle size. Thus, the replacement of Y with Zn or Cu on a molar basis after adding Zn- or Cu-nitrate salts resulted in a narrower silica network due to the smaller ionic radii of Cu (0.071 nm) and Zn (0.074 nm) compared to Y (0.090 nm). This narrower network led to a reduction in particle size [57–59]. Additionally, the specific surface area values ranged from 0.80 to 1.39 m^2^/g.

## 4. Discussion

The comprehensive analysis of the microspheres, including XRD, FT-IR, XPS, and SEM, provides a detailed understanding of their structural and compositional characteristics. XRD patterns, shown in Figure 1, confirm that all microsphere samples exhibit an amorphous structure, with no significant changes due to the incorporation of Zn and/or Cu dopants, indicating that the dopants did not affect crystallization. Similarly, Unal et al. [19] found that adding dopant elements did not result in any changes in the XRD peaks, further confirming the amorphous nature of the structure, as indicated by the XRD peaks. FT-IR spectra (Figure 2) validate the successful synthesis of YAS, with key peaks attributed to Si-O-Si bending vibrations, Si–O–Si vibrations, and various metal-oxide bond vibrations. Peaks at approximately 1627 cm^−1^ indicate adsorbed water. The bond vibrations of the Zn and Cu dopants could not be detected by FT-IR, necessitating XPS analysis, which confirmed the presence of these dopants. High-resolution XPS spectra (Figure 3b–f) revealed BEs consistent with Y, Al, Si, Zn, and Cu, validating the incorporation of Zn and Cu into the microspheres. SEM images (Figure 4) show that all samples maintain a spherical shape but exhibit an agglomerated morphology, similar to the findings of Ghahramani et al. [4], who synthesized spherical particles with an agglomerated morphology in their study. Particle sizes, ranging from 6 to 50 μm, and specific surface areas (0.80 to 1.39 m^2^/g) vary with the dopant type and amount, attributed to modifying the silica network by dopants. Replacing Y with Zn or Cu narrows the silica network, leading to smaller particle sizes. This integrated analysis underscores the successful synthesis and structural modifications of the microspheres, highlighting their potential for further applications in various fields.

## 5. Conclusion

1. Zn- and Cu-doped YAS glass microspheres were synthesized using a sol-gel-derived method in two stages. Initially, a sol was prepared and introduced into hot silicone oil with a syringe. The resulting solid gel spheres were subsequently dried and heat-treated at 600 °C for 5 h. XRD analysis confirmed that the microspheres possessed an amorphous structure and that the dopant elements did not influence the crystallization process.
2. FT-IR analysis identified Si–O–Si, Al–O–Al, and Y–O vibrations, confirming the presence of Y, Al, Si, and O in the microsphere structure. XPS analysis confirmed the successful incorporation of Cu and Zn into the YAS glass microspheres. Morphological analysis revealed an agglomerated spherical morphology. SEM analysis and particle-size measurements indicated that the average particle size ranged from 6 to 50 μm. Additionally, the specific surface areas of the microspheres varied between 0.80 and 1.39 m^2^/g.
3. Given that the particle size of YAS microspheres used in clinical practice ranges between 15 and 35 μm, our study identified that the 1Cu-YAS and 3Zn-YAS samples demonstrated optimal dopant amounts and types for use in radioembolization therapy. These antibacterial microspheres show promise in combating infections that may occur following radioembolization treatment.

## Figures and Tables

**Table 1:** Chemical compositions of the YAS glass microspheres.

Designation	Chemical composition (mol%)				
	Y_2_O_3_	SiO_2_	Al_2_O_3_	CuO	ZnO
YAS	17	64	19	0	0
1Cu-YAS	17	64	18	1	0
3Cu-YAS	17	64	16	3	0
1Zn-YAS	17	64	18	0	1
3Zn-YAS	17	64	16	0	3
1Cu2Zn-YAS	17	64	16	1	2
2Cu1Zn-YAS	17	64	16	2	1

**Table 2:** FT-IR spectra band assignment for the glass microspheres.

Band assignment	Wavenumber (cm^−1^)	References
Si-O-Si bending vibration	454	[38]
Si–O–Si vibration	535 and 1080	[39,40]
Aluminum oxide stress mode	515	[41]
Al-O-Al vibration	524 and 807	[42,43]
Y–O vibration	557	[44]
Adsorbed water molecules bending mode	1627	[40,45]

**Table 3:** XPS data in terms of spectral line and binding energy.

Element	Spectral line	Energy (eV)	References
Y	3d3/2	159.70	[46–48]
Y	3d5/2	157.61	[46–48]
Al	2p1/2	73.4 and 76.2	[49–51]
Al	2p3/2	72.3 and 74.4	[49–51]
Si	2p	102.1	[52]
Zn	2p3/2	1021.9	[53]
Zn	2p1/2	1044.9	[54]
Cu	2p3/2	932.2	[55]
Cu	2p1/2	952.6	[56]

**Table 4:** Average sizes and specific surface areas of the samples.

Designation	d_(0,9)_, (μm)	Specific surface area (m^2^/g)
YAS	23.53	1.25
1Cu-YAS	35.11	0.98
1Zn-YAS	48.49	0.80
3Cu-YAS	12.90	1.39
3Zn-YAS	29.64	1.04
1Cu2Zn-YAS	53.03	0.97
2Cu1Zn-YAS	51.18	0.94
